# Retro-Pharyngeal Abscess—Its Medical History and Treatment

**Published:** 1853-04

**Authors:** Charles M. Allen

**Affiliations:** Resident Surgeon of the New York Hospital.


### SELECTED ARTICLES.

### ARTICLE X.

*Retro-Pharyngeal Abscess—Its Medical History and Treatment.* By Charles M. Allen, M. D., Resident Surgeon of the New York Hospital.

(Continued from page 308.)

### DIAGNOSIS.

In the greater number of cases of retro-pharyngeal abscess occurring in adults, its existence can be distinguished from any other affection with but little difficulty, if the surgeon possesses the knowledge of, or suspects the possibility of its occasional formation. A local examination by the eye, or the finger, or by both, will generally reveal the true character of the disease. But this examination cannot always be satisfactorily effected. This is especially true in very young children, for obvious reasons, so that the recognition of the difficulty must be derived principally, if not wholly, from the rational signs. Complicated, as these sometimes are, with convulsions, or cerebral derangement, they are very liable to misinterpretation, the treatment being directed to these symptomatic complications, a suspicion of purulent collection behind the pharynx being never entertained.

The disease which most resembles *acute* pharyngeal abscess, and with which, therefore, it is most liable to be confounded, in the earlier period of life, is *croup*. By referring to the account of the phenomena, exhibited during the formation of the abscess, already recorded, it will be noticed that very many are entirely identical with those which occur in the course of an ordinary case of croup. There are, however, some well-marked points of difference between the two diseases, a careful attention to which will almost always guide to an accurate diagnosis. In the first place, the commencement of an attack of croup is very different from that of pharyngeal abscess. In the former, the peculiar crowing cough marks the beginning of the disease, in almost every instance, and more or less difficult and audible respiration is present from the first; in the latter, the crowing cough is never heard, and dyspnœa, increasing *gradually* in severity, is always and necessarily preceded by difficulty of deglutition, which is seldom urgent in croup. Again, in croup the difficulty of breathing is often very much relieved when the head is low, and is not increased by external pressure upon the larynx; in retro-pharyngeal abscess, on the contrary, the assumption of a horizontal position is immediately attended with so severe aggravation of the dyspnœa as to render its continuance impossible, without sacrifice of life; and pressure against the larynx from before, backwards, produces a similar effect, though in a less degree. The character of the voice, too, will very generally afford material assistance in the formation of a diagnosis between these diseases. In croup, it is at first hoarse, then weak and whispering, but always distinct; in pharyngeal abscess the peculiarity is well marked, and consists, as has already been intimated, in an obstructed nasal or guttural modification, it being very difficult to understand what the patient endeavors to communicate.

A specimen of a singular form of retro-pharyngeal abscess, the symptoms having been attributed to spasmodic croup, was exhibited at the first meeting of the Pathological Society of London, by Dr. Peacock, a brief report of which, on account of its peculiarity and interest, I will transcribe: "The sac of the abscess, which was the size of a small egg, was seen situated between the bodies of the upper cervical vertebræ and the back of the pharynx, not causing, however, much projection of the latter from its being flattened in front. In connection with the anterior surface of the sac there sprang a small cyst, forming a nipple-like prolongation into the pharynx, and completely closing the orifice of the glottis. It admitted the point of the little finger, and was freely movable, and perfectly translucent at its extremity and sides. The preparation was from an infant seven months old. The child had occasionally suffered from dyspnœa for three weeks, the symptoms having been very urgent for the last three days of its life. In the intervals of the dyspnœa, the respiration was natural, but the slightest exposure to cold, motion, or excitement, brought on a recurrence of the symptoms, which were attended, in inspiration, with a croupy sound."*

Another disease with which acute pharyngeal abscess may be confounded is *laryngitis*, accompanied with *œdema of the glottis and epiglottis.* The one can, however, be almost always distinguished from the other, if it be remembered that, in cases of the œdematous effusion, difficulty of breathing is most urgent during an *inspiration*, while, when this form of abscess is the cause, the dyspnœa is more continuous, being nearly the same during expiration as during inspiration. The sensation communicated to the finger in an examination by the mouth, is very different in the two cases. In the one, a soft pultaceous swelling is felt just at the base of the tongue, and the epiglottis, swollen and curled upon itself, is detected with comparative ease; in the other, the tumor is hard and elastic, situated *behind* the larynx; and the epiglottis may be felt or seen, entirely free from œdema. The more rapid progress of inflammation, and the total absence, or comparatively small degree, of dysphagia in laryngitis, will also aid in the formation of a diagnosis.

The *chronic* form of pharyngeal abscess has been mistaken for stricture of the œsophagus, for syphilitic ulceration of the throat, and for wry neck; but the error is directly traceable, in every instance, to ignorance or forgetfulness of the occasional occurrence of abscess in this region. Thorough exploration, then, is the great means by which this affection is to be distinguished from others occurring in the neighborhood, and when

* *Lond. and Edin. Month. Jour.* Oct., 1847.

made intelligently, will seldom fail to reveal the true cause of the dangerous conditions of deglutition and respiration above enumerated.

### PATHOLOGY.

The appearances presented in a post mortem examination of abscess in this region do not materially differ from those of abscess elsewhere. Enough has already been said in the course of this dissertation to indicate the seat of the affection, and to suggest its effects upon neighboring organs. The only peculiarity worthy of special notice is connected with the chronic variety. One of the causes, as we have seen, of this form, is scrofulous disease of the vertebræ; and it is an important fact that, while in the lumbar and dorsal regions the *bodies* of the bones are the seat of the disease, in the cervical region it ordinarily is confined to the *articular surfaces.* For this reason, in long-continued cases, in which the abscess has been opened, and the disease has not been arrested, dislocation of the vertebræ may take place, and death may, in this way, be the result of the laceration or compression of the spinal cord.

### PROGNOSIS.

From what has now been said with reference to this affection, it is right to infer, that in cases of its acute variety, if the difficulty is recognized, and the proper treatment employed, a favorable termination may be expected. If it passes unrecognized, and no spontaneous or accidental opening into its cavity be made, death is certain. In chronic cases, the result of treatment is not always so satisfactory. The existence of an abscess may be definitely ascertained, and immediate and essential relief afforded, by opening it, yet the disease of the bones may not be benefited by treatment, but continue to annoy the patient, until the dislocation just mentioned, or want of nutrition, terminate the case. In these chronic cases, too, as has been before noticed, the pus may extend downwards, in the loose areolar tissue behind the œsophagus, into the thorax, death being produced by an inflammation of the pleuræ and lungs, induced by this contact of purulent matter; and again, it is possible, that the prognosis may be modified by the formation of metastatic abscesses in one of the important internal organs, as the liver, or the lungs.

### TREATMENT.

From the enumeration which I have given of the different varieties of disease with which this form of abscess has been frequently confounded, it may be easily understood, how, in these cases, attempts have been made to effect a cure by emetics, purgatives, vesication, the application of leeches, phlebotomy, and other antiphlogistic remedies. In more than one instance, too, where the symptoms have been attributed to the existence of syphilitic ulceration of the throat, a strong solution of the nitrate of silver has been applied, locally, and the constitutional treatment of syphilis been persevered in, until the accidental rupture of the abscess, or death, revealed the nature of the affection. It is very evident, however, that it is only in the early stages of this disease, before the formation of the abscess has far progressed, that benefit is to be expected from the employment of these remedies, if indeed they are of service at any period of its progress. The process of suppuration, when once established, and deep seated, cannot be arrested simply by the use of antiphlogistic treatment, either general or local. Resort, therefore, to an operation, as a more direct and reliable means of relief, is inevitable, though this is, by no means, all that is required, to effect an entire recovery of the patient. Hence, the proper mode of treatment is necessarily divided into the *surgical*, or that adapted to a removal of the immediate cause of the urgent symptoms, and the *medical*, or that by which the patient is restored, as nearly as may be, to his original condition.

The surgical treatment of retro-pharyngeal abscess must obviously be the same in both the acute and chronic forms. I

shall therefore lay aside these distinctions, for the present, to be again referred to, when I come to consider the medical treatment required by each variety.

A temporary relief to the alarming dyspnœa may be afforded, by an opening into the larynx, the opening being made between the thyroid and cricoid cartilages, as in the usual operation of laryngotomy. This formidable operation would certainly never be resorted to, if the real cause of the symptom it is intended to relieve was ascertained, and it is mentioned here, not for the purpose of recommending its employment, in any case of this kind, but because, in some recorded cases, the practitioner supposing the case to be croup, the operation has been performed, the alleviation of the dyspnœa has been prompt, and apparently satisfactory, but a speedy return of all the fearful phonomena has given a fatal termination to the case, and the post mortem examination alone has discovered to the surgeon his error. The history of a case of this kind is given in the "*Archives Generales de Medicine*," tom. 57, p. 257.

Tracheotomy has also been adopted, as a remedy for the same symptom, but with the same ultimate result as laryngotomy, though somewhat longer delayed. Mr. Carmichael, of Dublin, was called to see a woman at the "Female Penitentiary," in that city, who presented the symptoms which I have described, as belonging to abscess behind the pharynx. The existence of such abscess was not detected, the diagnosis of acute laryngitis was decided upon, and the operation of tracheotomy was performed. The condition of the breathing was much amended, for the time, but on the second day she died, and a large abscess was found between the pharynx and the cervical vertebræ. It is very evident that these operations can only afford, even temporary, benefit to any other symptom than the dyspnœa. The difficulty of deglutition will, of necessity, remain as permanent as before. Thus, in the case just referred to, Mr. Carmichael says, on the day after the operation, "the patient now respired with ease, but the most alarming symptom was her total inability to swallow, even the smallest drop of liquid. She informed me, by writing, that she was starving, and felt the most acute pain from hunger."* These operations, therefore, at best, are only palliative in their effects, and are not to be relied upon in the treatment of this disease.

The only method of operating, from which permanent benefit can be expected, is that of a free opening into the cavity of the abscess, through which its contents may be discharged, and the immediate cause of the dyspnœa and dysphagia thereby be, partially at least, removed. This opening may be made in various ways. Mr. Fleming, in a somewhat extended article on "*Peculiar Affections of the Throat,*" &c., proposes, for this purpose, the use of a "pharynx trochar," which he has invented, and which he thus describes:—"It consists of a trochar about four inches long, one extremity of the canula being slightly curved, the other with a ring on the upper surface to receive the fore-finger; into this canula was passed a jointed stilette, with, at its opposite extremity, a ring for the thumb, and a movable screw to graduate the projection of its point."† The head of the patient being held by an assistant, this instrument, the point of the stilette being concealed, is guided upon the finger, backward, through the mouth to the anterior wall of the abscess; the stilette is then pressed forward to its limited mark, withdrawn, and the pus discharged through the canula. There is one good quality belonging to this instrument, and but one. It is, that, by the aid of the canula, the purulent collection may be evacuated, and the possible entrance of matter into the trachea be thus avoided. But notwithstanding this advantage, I would not recommend its use. I have already mentioned the great thickness and firmness of the posterior wall of the pharynx, in this disease, and have also spoken of its elasticity. If, now, the trochar be pressed against this firm elastic surface, the wall will yield before it, to a considerable distance: the stilette, being pushed forward, will pierce this wall, and by the sudden elastic spring, may, unless the utmost caution be observed, be driven against, or into, one of the vertebræ: caries may take place, and an abscess, acute in its character, and

* *Medico-Chirurgical Review*, vol. ii, p. 518.

† *Dublin Journal of Medical Science*, vol. xvii, p. 49

promising a speedy recovery, be converted into a chronic sinus, requiring months for its closure.

The same objections may be urged against the use of the "pharyngotome" of Petit, invented expressly for the opening of abscesses of the velum palati, and tonsils, but which has also been used for opening abscess behind the pharynx. The curved trochar of Sir Everard Home, for puncturing the bladder through the rectum, has also been recommended, but to that again the same objection is offered. Another very serious objection to the use of a trochar is, that the opening made by it is too small, and will close the moment the canula is withdrawn, so that the operation must either be frequently repeated, or the opening enlarged, by the aid of some other instrument.

The method which I would adopt, to effect the discharge of pus, is much more simple and effectual than those which have been mentioned, and may be described as follows. The head of the patient being firmly supported by an assistant, pass the forefinger of the left hand into the mouth, raise the velum palati, and press the point of the finger against the tumor. Then, with an ordinary scalpel, or bistoury, the blade being covered with adhesive plaster to within an half an inch of its extremity, let a free incision be made, in the median line, through the posterior wall of the pharynx, into the cavity of the abscess; withdraw the instrument, and the operation will be completed. The pain attending the operation will not be great, for the tension and thickening will have destroyed, to some extent, the sensibility of the part. By making a free incision at once, the necessity of repeating the operation is avoided, and the discharge of the purulent collection is more complete and satisfactory. No fistulous track is left to annoy the patient, and the recovery is more speedy, than when only a small opening is made. Should the position of the abscess be such, as to render it advisable that the incision be made at either side of the pharynx, particular care should be observed to avoid wounding the internal carotid artery, an accident which has occurred, in opening an abscess of the tonsil. The consequences of such an event are evident.

The abscess being thus opened and the dyspnœa relieved, our attention must now be turned to the subsequent treatment of the case. And here we find it necessary to recur to our former divisions of this abscess into the acute and chronic forms.

The treatment adapted to cases of acute pharyngeal abscess, after the purulent matter is discharged, is very simple, and demands but a moment's remark. It consists, in the external application to the neck of emollient and soothing remedies, such as poultices or warm fomentations, until the urgent symptoms shall have been entirely relieved, and the quantity of discharge from the abscess shall have been much diminished. When this result has been attained the recovery may often be facilitated, by the local employment of some astringent gargle. A very excellent combination for this purpose is the following:

| | |
|---|---|
| R. Bi boratis sodæ, . . . . | ʒ ij. |
| Tincturæ myrrhæ, . . . . | ℥ j |
| Syrupi simplicis, . . . . | ℥ ss. |
| Aquæ puræ, . . . . | ℥ viss. |
| | Misce. |

The condition of the general system is very generally such, as to call for the administration of tonics, and in many cases, even stimulants may be required. For the fulfilment of this indication, probably no better article of the materia medica can be recommended than the sulphate of quinine, and the most convenient mode of employing this remedy is in solution.

| | |
|---|---|
| R. Di-sulphatis quiniæ, . . . | gr. xvj. |
| Acidi sulphurici diluti, . . . | ʒ ss. |
| Syrupi zingiberis, . . . . | ℥ j. |
| Aquæ puræ, . . . . | ℥ vij. |
| | Misce. |

Cochleare magna sumenda ter in die.

This treatment, in connection with a nourishing diet, steadily persevered in, will, in almost every instance, restore the patient to his accustomed health, the time required for his complete recovery being subject to some variation, according to the effect which the disease may have produced upon the constitution.

In the medical treatment of chronic abscess behind the pharynx, our principal assistance must be derived from constitutional remedies, and these will be somewhat different, according as the cause is different, to which the formation of the abscess may be traced. If the patient be suffering from the effects of scrofulous or syphilitic cachexia, attention will be required to its relief. A detail of the mode of treatment adapted to these conditions would, however, be entirely foreign to the subject under consideration. So, too, the complication of disease of any of the cervical vertebræ, either recognized or reasonably suspected, will materially modify the character of the treatment, subsequent to the evacuation of the abscess. Here, then, as in the acute variety of the disease, so far as the treatment of the abscess itself is concerned, rest, tonics, and nourishing diet, are to be mainly relied upon to effect a restoration of the patient to his original condition.

I have thus, as I proposed at the commencement, attempted to collect and exhibit systematically, and as concisely as is consistent with clearness and completeness, the most prominent facts and phenomena attendant upon the progress and full development of retro-pharyngeal abscess; and to designate the modes, both surgical and medical, by which the disease may generally be conducted to a favorable termination.—*N. Y. Jour. Med.*

